# Entry inhibitors: New advances in HCV treatment

**DOI:** 10.1038/emi.2016.3

**Published:** 2016-01-06

**Authors:** Xi-Jing Qian, Yong-Zhe Zhu, Ping Zhao, Zhong-Tian Qi

**Affiliations:** Shanghai Key Laboratory of Medical Biodefense, Department of Microbiology, Second Military Medical University, Shanghai 200433, China

**Keywords:** antiviral target, entry factor, entry inhibitor, hepatitis C virus, hepatocyte

## Abstract

Hepatitis C virus (HCV) infection affects approximately 3% of the world's population and causes chronic liver diseases, including liver fibrosis, cirrhosis, and hepatocellular carcinoma. Although current antiviral therapy comprising direct-acting antivirals (DAAs) can achieve a quite satisfying sustained virological response (SVR) rate, it is still limited by viral resistance, long treatment duration, combined adverse reactions, and high costs. Moreover, the currently marketed antivirals fail to prevent graft reinfections in HCV patients who receive liver transplantations, probably due to the cell-to-cell transmission of the virus, which is also one of the main reasons behind treatment failure. HCV entry is a highly orchestrated process involving initial attachment and binding, post-binding interactions with host cell factors, internalization, and fusion between the virion and the host cell membrane. Together, these processes provide multiple novel and promising targets for antiviral therapy. Most entry inhibitors target host cell components with high genetic barriers and eliminate viral infection from the very beginning of the viral life cycle. In future, the addition of entry inhibitors to a combination of treatment regimens might optimize and widen the prevention and treatment of HCV infection. This review summarizes the molecular mechanisms and prospects of the current preclinical and clinical development of antiviral agents targeting HCV entry.

## Introduction

Hepatitis C virus (HCV) belongs to the family *Flaviviridae* and infects more than 180 million people worldwide. HCV infection is considered as a major public health problem and consumes millions of dollars in medical expenses every year.^1,2^ HCV has a total of seven identified genotypes, with more than 50 subtypes and millions of quasispecies. The high variability and complexity of the virus make it difficult to manufacture effective prophylactic or therapeutic vaccines to prevent the pathogen from spreading. Approximately 70% of acutely infected patients will ultimately develop chronic infections despite the implementation of advanced medical care and intervention.^3^ Due to its biological characteristics, HCV infection is one of the leading causes of liver-associated diseases, such as cirrhosis, steatosis, and hepatocellular carcinoma, whose end-stage patients require liver transplantation to stay alive.^4^ Unfortunately, the reinfection of a graft is difficult to avoid due to the lack of preventive strategies.^5^

The previously recommended treatment for HCV infection was a combination therapy consisting of PEGylated interferon alpha and ribavirin.^3^ In recent years, HCV treatment has undergone a groundbreaking evolution. Direct-acting antivirals (DAAs), such as protease inhibitors (boceprevir or telaprevir in 2011), have revolutionized the current status of HCV treatment. Triple-combination therapy improves sustained virological response (SVR) rates in naive genotype 1 patients by more than 70%. However, the two first-generation protease inhibitors that are typically used easily lead to the development of drug-resistant variants, and concomitant adverse reactions such as fatigue or anemia unavoidably reduce patient compliance with the regimen.^4,6,7^ A second-wave first-generation protease inhibitor, simeprevir, and a nucleotide analog, sofosbuvir, were approved by the United States in 2013 via the FDA and by Europe in 2014 for the treatment of hepatitis C (HC).^7,8,9^ In October 2014, the use of ledipasvir/sofosbuvir was approved by the FDA, and in December, an interferon-free regimen including an ombitasvir/paritaprevir/ritonavir combination tablet and dasabuvir was also approved for the treatment of genotype 1 patients.^10,11,12,13,14,15^ A number of other DAAs and host-targeted agents (HTAs) are undergoing clinical trials. Daclatasvir is an NS5A inhibitor and is currently being evaluated in an advanced clinical trial as a component of a combination therapy.^16^ In fact, the combination of daclatasvir and asunaprevir (an HCV NS3/4A protease inhibitor) has been approved for the treatment of genotype 1 patients in Japan.^16^ The future of HCV therapy is likely to be consist of interferon-free regimens with pan-genotypic activity, higher antiviral efficiencies, shorter treatment durations, and fewer adverse reactions. The emerging novel antivirals should optimize the treatment options, especially for difficult-to-treat patients, such as those who are suffering from advanced liver diseases or other co-infections and who have poor response rates to current regimens.^17,18^

**Figure 1:**
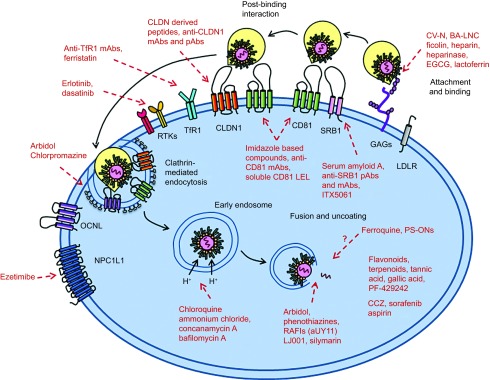
HCV entry into hepatocytes and antiviral agents targeting entry factors. The HCV lipoviral particle (LVP) is recruited and binds to glycosaminoglycans and low-density lipoprotein receptor on host cells. After binding, the virions interact with a series of entry factors. SRB1 plays a role in both binding and post-binding. CD81 interacts with HCV E2, forms a complex with claudin-1 (CLDN1), and mediates HCV movement to the tight-junction areas. This process is regulated by the receptor tyrosine kinase (RTK) family, including epidermal growth factor receptor (EGFR) and ephrin receptor A2 (EphA2). The virions internalize into host cells by clathrin-mediated endocytosis. Transferrin receptor 1 (TfR1) facilitates viral entry after CD81, possibly during HCV particle endocytosis. Niemann Pick C1-like 1 (NPC1L1) plays an important role in cholesterol transportation and is a cofactor for HCV entry during post-binding steps. Low pH-dependent membrane fusion between endosome and HCV particle. Red words and lines indicate the antiviral agents targeting different stages and factors of HCV entry.

HCV entry represents the beginning of viral infection, which is highly orchestrated and essential in initiating viral infection and spread. HCV entry includes the initial recruitment and attachment of the virus to hepatocytes, post-binding interactions with host entry factors, clathrin-mediated endocytosis, and a final low pH-triggered membrane fusion to release viral RNA into the cytosol (Figure 1). The blocking of viral entry can efficiently eradicate HCV infection at the very first step, before viral genomes start to emerge, and might prevent cell-to-cell transmission, which is also required for viral spread. The current antiviral agents that are on the market or being evaluated in clinical trials mainly focus on targeting HCV nonstructural protein maturation or viral RNA synthesis. Although the currently used cocktail therapy is believed to cure more than 90% of infected patients, the appearance of viral resistance, null responders or treatment failure, superimposed with the adverse effects caused by the drugs, is still a major limitation that must be resolved.^19^ As an RNA virus, HCV very easily develops a resistance to antiviral treatments due to its error-prone replication property. Most entry inhibitors target host components, such as receptors or key enzymes, which are required for HCV entry and definitely have high genetic barriers to resistance due to their conserved nature. Therefore, these inhibitors tend to not only have pan-genotypic activity against virus infection but also have a greater risk of simultaneously causing cellular toxicity. Moreover, as the initial step of the viral life cycle has been blocked, entry inhibitors also have prophylactic properties. Because entry inhibitors target a different stage of the viral life cycle than the currently used anti-HCV drugs, they might have a synergistic effect when combined with the current regimens, especially with DAAs, providing multiple novel targets and new insight into antiviral strategies and complementary existing antiviral interventions, such that viral clearance may finally be achieved. This article reviews the entry inhibitors that are currently in development.

## HCV entry into hepatocytes

### Initial low-affinity attachment and binding

*In vivo*, circulating HCV particles reach the basolateral surfaces of hepatocytes, where the virus first binds to several receptors with low affinity, allowing it to become concentrated on the host cells' surfaces to enable subsequent interactions with other essential entry factors (Figure 1). These attachment factors include glycosaminoglycans (GAGs) on heparan sulfate (HS) and low-density lipoprotein receptor (LDLR). Both of these receptors are able to interact with viral envelope proteins and apoE on lipo-virion particles (LVPs).^20,21,22^ HCV exists as LVP with LDL and VLDL in the circulatory systems of chronically infected patients. Recent studies have demonstrated that LDLR plays an important role in HCV attachment to target cells.^23^ The knockdown of this receptor by small interfering RNA (siRNA) potently reduces virus infectivity, and a soluble form of LDLR can impair virus binding by interacting with HCV particles.^24^ Although this receptor is dispensable for lipid/cholesterol-free HCVpp entry, productive HCV infection, including viral entry and replication, requires the involvement of LDLR.^24,25^

The lectin cyanovirin-N (CV-N) is a type of carbohydrate-binding agent that has potent antiviral activity against HCV. This small compound impairs virus binding by interacting with viral envelope glycoproteins at their high-mannose oligosaccharides (Table 1).^26^ Recent studies have identified boronic acid-modified lipid nanoparticles (BA-LNC) as potent inhibitors of HCV entry through a mechanism similar to that of lectins. BA-LNCs could be used as a pseudolectin-based therapeutic agent to develop novel HCV entry inhibitors.^27^ Ficolins are a type of serum protein related to collectins.^28^ Neutralizing concentrations of L-ficolin can be found in the sera of HCV-infected patients. Additionally, a recent study shows that recombinant human L-ficolin can neutralize HCV particles directly and inhibit virus attachment by neutralizing the viral glycoproteins E1 and E2.^29^ Heparin is a structural analog of HS that can competitively inhibit virus binding to host cells. A series of heparin-derived molecules are undergoing evaluation for their potential to serve as anti-HCV agents.^21,90^ Heparanase, an enzyme that degrades HS on host cell surfaces, can also impede both HCV E2 and HCVcc binding to host cell surfaces (Table 1).^21^

Epigallocatechin gallate (EGCG) and its derivatives are natural polyphenol compounds that are abundant in green tea extracts and have long been considered to regulate lipid metabolism, thereby having the potential to affect a variety of diseases.^91^ Studies suggest that EGCG and its derivatives impair virus binding to the host cell by interfering with virion E1/E2 function and simultaneously blocking cell-to-cell transmission *in vitro* (Table 1).^30,31,32^ Additionally, limited sampling estimates of EGCG in HCV patients suggest that a single oral dose of up to 400 mg of this green tea extract is safe and well tolerated.^92^ Additional experiments are needed to evaluate the possibility of using this compound as a candidate for anti-HCV therapy.

Lactoferrin (LF) is an iron-binding glycoprotein that belongs to the transferrin family; it is abundant in milk and most biological fluids.^93^ The antiviral activity of LF is relatively well understood. LF is thought to function by directly interfering with HCV particles to prevent their attachment to host cells both *in vitro* and *in vivo*.^33^ Bioactive peptides, such as the N-lobe or C-lobe of LF, also inhibit virus infection.^35^ Among all species, camel lactoferrin (cLF) shows the most effective antiviral property and is now being evaluated in a clinical trial (Table 1).^34^

The p7 is a polypeptide of the HCV-encoded protein in the endoplasmic reticulum membrane and is essential for infectious viral production *in vivo*.^94,95^ A recent study revealed that a p7 ion channel-derived peptide H2-3 potently inhibits HCV entry by directly affecting virus binding to the cell surface and interfering with the virus–host interaction (Table 1).^36^

### Post-binding interactions with specific entry factors

Subsequent to virus binding, LVPs start to form contacts with a series of entry factors on host cells. Targeting these relatively conserved factors, which are indispensable for the early life cycle of the virus, builds up genetic barriers against antiviral agents. HCV entry requires several host factors, including the tetraspanin molecule CD81, scavenger receptor class B type 1 (SRB1), the tight junction (TJ) proteins claudin-1 (CLDN1) and occludin (OCLN), transferring receptor 1 (TfR1), the receptor tyrosine kinases (RTKs) epidermal growth factor receptor (EGFR) and ephrin receptor A2 (EphA2), and Niemann-Pick C1-like 1 (NPC1L1) cholesterol uptake receptor (Figure 1).

CD81 was the first of these factors to be identified and is the best understood HCV entry factor. It is a ubiquitously expressed, 26 kDa transmembrane protein that consists of a small extracellular loop (SEL) and a large extracellular loop (LEL).^96,97,98^ The CD81 LEL is believed to interact with the HCV E2 protein, which contributes directly to HCV infection. Imidazole-based compounds simulate the D-helix of CD81 and are relatively safe small molecule inhibitors of HCV. They selectively abrogate the function of CD81 during HCV entry, while the remaining physiological functions of CD81 are basically preserved (Table 1).^37^ Specific anti-CD81 monoclonal antibodies (mAbs), such as JS-81 or the newly developed K04, counteract E2-CD81 interactions, interfering with viral entry during a post-binding process and inhibit HCV infection in humanized mice (Table 1).^38,39,40,41,42,43^ A soluble recombinant form of CD81 LEL shows effective anti-HCV activity and is able to inhibit the entry of HCVpp, HCVcc, and serum-derived HCV, as well as HCV infection, *in vivo* (Table 1).^42,44,45,46,47,48^ However, because CD81 is widely distributed in all tissues, the toxicity issues that are associated with using CD81-based antibodies or compounds should be evaluated carefully.

SRB1 is a horseshoe-shaped glycoprotein that is closely related to lipid metabolism. SRB1 binds diverse lipoproteins, including HDL, LDL, and oxLDL and plays key roles in bidirectional cholesterol transport, possibly modulating HCV entry into host cells.^99,100^ The extracellular loop of SRB1 interacts with the HCV E2 HVR1 region and is required for viral entry during both binding and post-binding steps.^51,101^ Serum amyloid A (SAA) is an acute-phase protein that is produced by the liver.^102,103^ There is a close relationship between SAA and HDL in modulating HCV infectivity.^50^ SRB1 binds to and internalizes SAA, and SAA inhibits HCV entry by interacting with the virus (Table 1).^49,50^ Antibodies targeting SRB1 inhibit virus infection and spread both *in vitro* and in a humanized mouse model (Table 1).^51,52,53,54^ The preclinical compound ITX5061 is a small-molecule antiviral that impedes the uptake of HDL through SRB1, thus blocking the uptake of viral particles.^55,56^ An *in vitro* study indicated that ITX5061 functions synergistically with DAAs, making it a promising candidate for future combination therapy.^57^ This compound has just finished evaluation in a phase Ib study and is now undergoing a phase II clinical trial in HCV-positive patients (Table 1).^58^

CLDNs and OCLNs are components of TJs. CLDN1 is believed to form a complex with CD81 and to contribute to efficient HCV internalization.^104,105,106^ The expression of CLDN1, CD81, and SRB1 can confer HCVpp entry into HEK293 cells.^107^ CLDN1 is highly expressed in hepatocytes, making it an ideal and promising target for the development of specific prophylactic antiviral agents.^108^ A human CLDN1-derived peptide (CL58) was screened out from an overlapping peptide library and was confirmed to have antiviral activity during a late post-binding step without affecting TJ integrity (Table 1).^59^ CLDN1 mAbs and pAbs show potent inhibitory effects on HCV infection, probably because they can neutralize E2-CD81-CLDN1 associations with low toxicity in primary human hepatocytes (PHHs) and humanized mice (Table 1).^60,61,62^ However, a recent study has suggested that broad CLDN tropism permits its escape from CLDN1 Abs because the virus can utilize CLDN6 proteins in the same cell, providing new insight into CLDN usage during HCV infection.^109^ OCLN is also a key entry factor for HCV, as the expression of human OCLN and CD81 in mouse liver leads to viral permissivity in this originally non-susceptible animal model, while the silencing of this receptor perturbs HCV entry during a late post-binding step.^110,111,112^ A recent study found that the overexpression of miR-122 can decrease HCV entry by downregulating OCLN.^113^

EGFR and EphA2 are two well-understood RTKs and have recently been identified as host cofactors of HCV entry by a functional siRNA kinase screen.^63^ These two kinases are highly expressed in human liver, and their specific inhibitors erlotinib (an EGFR inhibitor) and dasatinib (an EphA2 inhibitor), two clinically approved anticancer compounds, significantly impair viral entry in both polarized hepatoma cells and PHHs; erlotinib has also been effective in human-liver chimeric mice (Table 1).^63^ RTKs modulate viral entry at post-binding steps by interfering with CD81–CLDN1 complex association and blocking cell-to-cell transmission, all of which makes these two RTKs promising targets for developing anti-HCV agents, especially for the prevention of graft reinfection in chronic HCV patients who must undergo liver transplantation.^114^ Still, the specific efficacy and safety of using the currently licensed inhibitors of RTKs in HCV treatment requires further clinical evaluation.

Clinical observational data suggest that an iron metabolic disorder might occur in HCV-positive patients.^115,116,117^ Transferrin receptor 1 (TfR1), an iron uptake receptor, is widely expressed in mammalian cells, including hepatocytes, and its trafficking protein (TTP) is involved in HCV entry. Both a specific anti-TfR1 mAb and a TfR1 inhibitor, ferristatin, impede HCV infection without affecting viral RNA replication when treatment is applied before virus incubation (Table 1).^64^ Kinetic experiments suggest that TfR1 facilitates HCV entry at a post-binding step, most likely after CD81.^64^ Further studies are needed to identify the functional domain of TfR1 that is used during HCV entry, which will enable the development of specifically targeted antiviral agents. However, HCV cell-to-cell transmission is not completely reduced after treatment with either the anti-TfR1 antibody or the TfR1 inhibitor, indicating that a different potential mechanism might be involved in this process.

NPC1L1 is a 13 transmembrane cholesterol transport receptor that is highly expressed on the apical surfaces of human enterocytes and the canalicular membranes of human hepatocytes.^118,119^ The main function of it is to modulate cholesterol homeostasis in the body.^120^ A correlation exists between the NPC1L1 level and HCV infection.^66^ Specific antibodies, especially ones that target the NPC1L1 large extracellular loop 1 (LEL1), effectively eradicate HCV entry in a manner similar to that of CD81 antibodies, indicating the potential action mode of this receptor.^65^ Ezetimibe is an FDA-approved NPC1L1 antagonist that is clinically used to treat hypercholesterolemia. The application of ezetimibe inhibits HCV entry and cell-to-cell transmission *in vitro*, while *in vivo,* this drug delays the establishment of genotype 1 HCV infections in severe combined immunodeficiency (SCID) mice with humanized hepatocytes (Table 1).^66^ The therapeutic window in humans has not yet been determined.

### Clathrin-mediated viral endocytosis and membrane fusion

After interacting with a series of receptors, HC virions are internalized into cells via a highly coordinated process, most likely through clathrin-mediated endocytosis together with some cell entry factors, such as CD81 and CLDN1.^70^ The trafficking of the CD81–CLDN1 receptor complex promotes simultaneous virus internalization and fusion.^60,104,106^ During this process, the internalized vesicles form early and late endosomes, which are then prepared for the subsequent virion-cell fusion process.^69^ The use of either siRNA molecules targeting the clathrin heavy chain or the specific inhibitor chlorpromazine, which effectively interferes with clathrin-coated pit formation, impairs HCVpp entry and HCVcc infection in host cells (Table 1).^67^ Arbidol is an indole-derivative molecule that is licensed as an anti-influenza drug in China and Russia.^121,122^ This broad-spectrum antiviral uses several approaches to inhibit HCV cell entry. One of its antiviral mechanisms involves affecting clathrin-mediated endocytosis by impairing clathrin-coated pit release and dynamin-2-induced membrane scission (Table 1).^68^

Membrane fusion between the virus and host cell is the final step before the viral genome is released into the cytosol to start translation and replication.^123^ The envelope proteins are primed by virus–receptor interactions (most likely CD81) to become sensitive to low pH, and the fusogenic conformational changes of the related peptides are activated by the acidic environment and proper temperature of the endosomal lumen.^71,90,124^ Lipids, including cholesterol and sphingomyelin (SM), are essential components that facilitate HCV fusion.^125,126^ Studies have shown that the addition of cholesterol enhances the fusion efficiency of the HCV particle. Additionally, among virions of different densities, the lowest-density particle, HCVcc, exhibits the highest specific fusogenicity.^127^

HCV fusion inhibitors are basically characterized into the following three groups. The first group targets the acidification-triggering mechanism of virion–cell membrane fusion. Currently available acidification inhibitors include concanamycin A and bafilomycin A, two potent inhibitors of vacuolar ATPases (Table 1).^69^ Chloroquine and ammonium chloride are also considered to disturb endosome acidification and to suppress the occurrence of membrane fusion in a dose-dependent manner (Table 1).^70,71^ The second group focuses on the lipid compositions of both virus and host cells, which are indispensable throughout the process of virus fusion. In addition to its inhibitory effect on viral endocytosis, the indole-derivative arbidol also inhibits HCV membrane fusion, most likely via a dual-binding mode that involves aromatic residues in viral glycoproteins and phospholipids at the membrane–water interface, thus impeding the required conformational changes of fusion peptides during virus fusion (Table 1).^72^ Phenothiazines are small-molecule compounds containing nitrogen and sulfur tricyclic structures. Recently, three phenothiazine compounds have been identified as potent HCV entry inhibitors (Table 1); they suppress virion–cell membrane fusion by incorporating into target membranes and increase the fluidity of cholesterol-rich membranes, destabilizing the pre-fusion state of the virus.^73^ Rigid amphipathic fusion inhibitors (RAFIs) are a class of synthetic rigid amphiphiles that are similar to phospholipids. RAFIs can interact with envelope lipids and increase the activation barrier of viral proteins, leading to the blockage of increased negative curvature during the initial viral fusion stage (Table 1).^74^ AUY11 is a representative arabino-based RAFI.^75^ LJ001 is a small-molecule compound that specifically intercalates into viral membranes to inactivate the virion from a pre-fusion state, thereby blocking virus fusion (Table 1).^76^ Silymarin is a compound mixture of several flavonolignans and flavonoid taxifolins; it inhibits HCV infection both *in vitro* and *in vivo* in a similar pattern to that of arbidol (Table 1).^77,78^ The third group of fusion inhibitors includes several compounds with unclear mechanisms, including ferroquine, a chloroquine analog, and phosphorothioate oligonucleotides (PS-ONs), which act as amphipathic DNA polymers.^79,80^ PS-ONs inhibit HCV infection both *in vitro* and *in vivo*, possibly at the fusion step (Table 1).

Some natural, plant-derived compounds, such as the flavonoid ladanein, the terpenoids saikosaponin, oleanolic acid, tannic acid or gallic acid, or the small molecule SKI-1/SIP inhibitor PF-429242, have antiviral activities against HCV during an early stage of viral infection (Table 1).^81,82,83,84,85,86^ However, their exact antiviral mechanisms and potential applications in clinical practice require further investigation and evaluation.

Moreover, several FDA-approved drugs that have already been qualified for clinical application have shown antiviral activities against HCV infection. Chlorcyclizine HCl (CCZ) is an over-the-counter anti-histamine drug for allergy symptoms. CCZ blocks late-stage of HCV entry before viral replication and is now undergoing a phase Ib clinical trial.^87^ Sorafenib is a multi-kinase inhibitor that has been approved for the treatment of hepatocellular carcinoma.^128^ A recent study shows that sorafenib inhibits both HCV entry and production and affects CLDN1 expression and localization.^88^ Aspirin, a commonly used analgesic and anti-platelet drug, blocks HCV entry by downregulating CLDN1 (Table 1).^89^ These drugs are relatively accessible and affordable agents with an established clinical safety profile, thereby becoming promising candidates for drug repurposing for the treatment HCV infection.

## Conclusions and perspectives

The unveiling of the molecular mechanisms of HCV entry in recent years has largely promoted the development of entry inhibitors targeting different stages of the early viral life cycle. Because viral entry is essential for the initiation, spread, and maintenance of HCV infection, great potential and numerous prospects exist for entry inhibitors to be applied as members of cocktail therapy in future HCV treatments. Although newly identified entry inhibitors have emerged continuously in recent years, most of them still remain in the *in vitro* stages of testing. Until now, only a few have entered clinical trials, including the most advanced entry inhibitor, ITX5061, which targets the host entry factor SRB1 to interfere with virus infection. This compound is currently undergoing phase II clinical trials and appears to be a promising option for future combination therapy.^9,56,63^ Regardless of its outcome, ITX5061 provides a good example for the study and development of entry inhibitors. Moreover, several compounds with favorable anti-HCV potencies have already been approved to treat other diseases in the clinic and provide a good method of screening for novel entry inhibitors of HCV. Table 1 summarizes the targets and developmental stages of current antiviral agents during the HCV entry process.

Unlike the currently marketed anti-HCV DAAs, which target viral proteins of high variability, most entry inhibitors are HTAs with high genetic barriers, which are valuable features for avoiding viral escape, due to their conserved nature. Because all of the major HCV genotypes are believed to enter host cells via the same cellular pathways, the antiviral activities of most entry inhibitors tend to be genotype-independent.^129^ Moreover, entry inhibitors are also bestowed with the unique advantage of preventing cell-to-cell transmission as long as their antiviral targets cover common factors in both cell-free and cell-to-cell viral spread.^130^ Additionally, such drugs will help end-stage HCV patients resist graft reinfection after undergoing liver transplantation. A series of novel antiviral agents is currently being tested in *in vitro* assays or *in vivo* animal models. Some of these agents have entered the clinical stage for evaluation in patients. The satisfactory outcomes of this class of entry inhibitors should complement current treatment approaches and lead to more efficient, economical and better tolerated options for HCV patients, especially difficult-to-treat patients.

## Figures and Tables

**Table 1:** The process of viral entry and targets for antiviral agents with their development stage

Process of entry	Target	Representatives of compounds	Developmental stage	References
Attachment		Lectin cyanovirin-N	Cell culture	^26^
		BA-LNC	Cell culture	^27^
		Ficolin	Cell culture	^28,29^
		Heparin and heparin-derived compounds	Cell culture	^21,28^
		Heparanase	Cell culture	^21^
		EGCG and its derivatives	Cell culture	^30,31,32^
		Lactoferrin	Phase I	^33,34,35^
		A p7 ion channel-derived peptide H2-3	Cell culture	^36^
Post-binding interactions with entry factors	CD81	Imidazole-based compounds	Cell culture	^37^
		Anti-CD81 mAbs	Mouse model	^38,39,40,41,42,43^
		Soluble CD81 LEL	Cell culture	^42,44,45,46,47,48^
	SRB1	Serum amyloid A	Cell culture	^49,50^
		Anti-SRB1 pAb and mAb	Mouse model	^51,52,53,54^
		ITX5061	Phase I/IIa	^55,56,57,58^
	CLDN1	Anti-CLDN1 peptides	Cell culture	^59^
		Anti-CLDN1 pAb and mAb	Mouse model	^60,61,62^
	EGFR	Erlotinib	Phase I/IIa	^63^
	EphA2	Dasatinib	Cell culture	^63^
	TfR1	Anti-TfR1 mAbs	Cell culture	^64^
		Ferristatin	Cell culture	^64^
	NPC1L1	Anti-NPC1L1 mAbs	Cell culture	^65^
		Ezetimibe	Mouse model	^66^
Clathrin-mediated endocytosis		Chlorpromazine	Cell culture	^67^
		Arbidol	Cell culture	^68^
Fusion and uncoating	Endosome acidification	Concanamycin A	Cell culture	^69^
		Bafilomycin A	Cell culture	^69^
		Chloroquine	Cell culture	^70,71^
		Ammonium chloride	Cell culture	^70,71^
	Lipid composition of virus or host cell	Arbidol	Cell culture	^72^
		Phenothiazines	Cell culture	^73^
		RAFIs (aUY11)	Cell culture	^74,75^
		LJ001	Cell culture	^76^
		Silymarin	Cell culture	^77,78^
	Unclear mechanism	Ferroquine	Cell culture	^79^
		PS-ONs	Mouse model	^80^
Natural compounds and small molecules		Flavonoids, terpenoids, tannic acid, gallic acid, PF-429242	Cell culture	^81,82,83,84,85,86^
FDA-approved drugs		CCZ, sorafenib, aspirin	Phase Ib Cell culture	^87,88,89^
